# Cost-effectiveness of adding vaccination with the AS04-adjuvanted human papillomavirus 16/18 vaccine to cervical cancer screening in Hungary

**DOI:** 10.1186/1471-2458-12-924

**Published:** 2012-10-30

**Authors:** Zoltán Vokó, László Nagyjánosi, Zoltán Kaló

**Affiliations:** 1 Department of Health Policy & Health Economics, Institute of Economics, Faculty of Social Sciences, Eötvös Loránd University, 1117, Budapest, Pázmány Péter sétány 1/a, Hungary; 2 Syreon Research Institute, 1146, Budapest, Thököly út, 119, Hungary; 3 National Institute for Health Development, 1094, Budapest, Nagyvárad tér 2, Hungary

**Keywords:** Cervical cancer, Human papillomavirus, Vaccine, Cervarix, Hungary, Cost-effectiveness

## Abstract

**Background:**

The cervical cancer screening program implemented in Hungary to date has not been successful. Along with screening, vaccination is an effective intervention to prevent cervical cancer. The aim of this study was to assess the cost-effectiveness of adding vaccination with the human papillomavirus 16/18 vaccine to the current cervical cancer screening program in Hungary.

**Methods:**

We developed a cohort simulation state-transition Markov model to model the life course of 12-year-old girls. Eighty percent participation in the HPV vaccination program at 12 years of age was assumed. Transitional probabilities were estimated using data from the literature. Local data were used regarding screening participation rates, and the costs were estimated in US $. We applied the purchasing power parity exchange rate of 129 HUF/$ to the cost data. Only direct health care costs were considered. We used a 3.7% discount rate for both the cost and quality-adjusted life years (QALYs). The time horizon was 88 years.

**Results:**

Inclusion of HPV vaccination at age 12 in the cervical cancer prevention program was predicted to be cost-effective. The incremental cost-effectiveness ratio (ICER) of adding HPV vaccination to the current national cancer screening program was estimated to be 27 588 $/QALY. The results were sensitive to the price of the vaccine, the discount rate, the screening participation rate and whether herd immunity was taken into account.

**Conclusions:**

Our modeling analysis showed that the vaccination of 12-year-old adolescent girls against cervical cancer with the AS04-adjuvanted human papillomavirus 16/18 vaccine would be a cost-effective strategy to prevent cervical cancer in Hungary.

## Background

Since the introduction of human papillomavirus (HPV) vaccines, both primary and secondary preventive (i.e., screening) measures have been available to prevent cervical cancer. Health policy makers need to answer the following question: what is the most effective and cost-effective strategy for cervical cancer prevention in a certain country?

Although there is insufficient evidence regarding the effectiveness of cervical screening (Pap-smear) among vaccinated women [1], most policy recommendations advocate continuation of the screening programs in vaccinated women because current vaccines do not provide protection against all oncogenic types of HPV and because the overall vaccine efficacy against cervical cancer is less than 100% [2-4].

Thus, the relevant question from the health policy perspective is whether introducing a cervical cancer vaccination program for adolescents in parallel with the screening program is cost-effective.

The organized cervical screening program was launched for women aged 25–65 in 2003 as part of the National Public Health Program in Hungary aiming to target those women who otherwise would not use the service. Unfortunately, the organized screening program had only a small positive effect on the percentage of the population that participated in regular screening [5,6]. Improving the effectiveness of the cervical cancer prevention program is on the agenda of Hungarian health policy makers. We have recently developed a health economic model to study the cost-effectiveness of different possible screening strategies [7].

In addition to the development of the current screening program, policy makers need to decide whether to introduce vaccination against cervical cancer. The vaccination of adolescent girls is the most likely form of vaccination to be added to the program. There are two vaccines available for preventing cervical cancer. One of these is Cervarix^TM^, an AS04-adjuvanted bivalent vaccine against HPV types 16 and 18 produced by GlaxoSmithKline. The second vaccine, Gardasil ^TM^, is active against HPV types 6, 11, 16 and 18. The purpose of this study was to assess the cost-effectiveness of adding vaccination with the AS04-adjuvanted human papillomavirus 16/18 vaccine (Cervarix^TM^) at age 12 to the current national cervical screening program. We have not considered screening methods other then the prevailing one in the national cervical cancer screening program.

## Methods

We developed a cohort simulation state-transition Markov-model using Microsoft Excel software. The model estimates the life course of 12-year-old girls.

**Figure 1:**
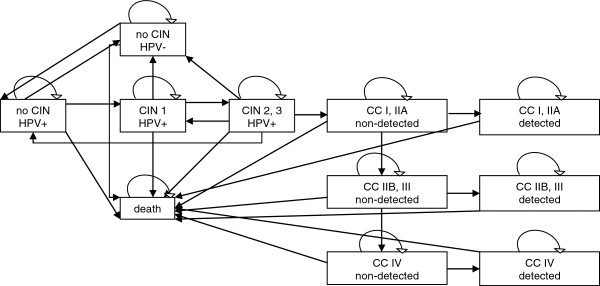
**Health states used in the disease progression model.** CIN: cervical intraepithelial neoplasia, CC: cancer, HPV: human papilloma virus.

The disease progression part of the cost-effectiveness model was based on a previously published model (Figure 1) [7-9]. The boxes represent health states, and the arrows represent transition routes between the health states. We calculated the transition probabilities for a cycle length of one month assuming a constant incidence rate for one cycle (see these incidences in Additional file 1). The time horizon of the model was 88 years, i.e., the accumulated costs and QALY differences were estimated at age 99 years. Data on quality-of-life (QoL) weights were taken from a previously published Hungarian model assessing the cost-effectiveness of different screening strategies (Table 1) [7].

**Table 1:** Quality of life weights* corresponding to different cancer stages

	**Non-detected**	**Newly detected**	**Treated**
CC I-IIA	1.0 (0.1000)	0.68 (0.0680)	0.95 (0.0950)
CC IIB-III	0.95 (0.0950)	0.56 (0.0560)	0.75 (0.0750)
CC IV	0.9 (0.0900)	0.48 (0.0480)	0.60 (0.0600)

The numbers in brackets are the standard errors applied for the distributions in the probabilistic sensitivity analysis.

*The age specific quality of life weights are multiplied with these weights.

The discount rates of cost and quality of life years (QALY) are user inputs in the model. For the base case, we used 3.7% for both discount rates, which is in line with the recommendation of the working group on discount rates in health economic analysis lead by the National Health Fund. We also provide the results calculated with a discount rate of 5%, which is specified in the current Hungarian guidelines for health economic evaluations [10]. Our analysis was conducted from the public health care payer’s perspective.

### Screening

An organized cervical cancer screening program was launched for women aged 25 to 65 years in 2003 as part of the National Public Health Program. Women were invited to undergo cervical screening every 3 years. The method of screening followed the longstanding Hungarian professional tradition of opportunistic screening; it included Papanicolaou test and colposcopic examination performed by gynecologists in outpatient services located in cities. Regarding the procedures following the screening test, if a person has a Papanicolaou 3 (P3) cytology result, she receives a combination of local anti-inflammatory treatment and the cytology is repeated within 2 weeks. If the subsequent result is P3 or worse again, then conization is offered just like for those who had a P4 or P5 result initially. If the subsequent result after an initial P3 cytology is P2, then the cytology is repeated again in 6 months. If this result is Papanicolaou 1 (P1) or Papanicolaou 2 (P2), then the patient returns to the regular screening regimen. If the result is worse than P2, conization is offered.

In line with the current practice, screening was assumed to occur every 3 years in the model. The proportion of women starting and stopping cervical screening can be set in the model at each screening period, allowing great flexibility of modeling different uptakes of screening at different ages. In the default setting, we set the proportion of women starting and stopping screening at each round of the screening in such a way that the participation rates were in line with the current age-specific participation rate figures (Table 2) [11]. The data on the test characteristics of the combined test of cytologic and colposcopic examinations were taken from our model for cervical screening (Table 3) [7].

**Table 2:** Proportion of the population screened within three years by age group

**Age group (years)**	**Percentage screened**
12-17	0%
18	32%
21	52%
24	62%
27	65%
30	66%
33	66%
36	63%
39	61%
42	56%
45	53%
48	51%
51	50%
54	49%
57	40%
60	36%
63	32%
66	27%
69	22%
>69	0%

**Table 3:** The default setting of the distribution of the screening test results according to the disease stages

	**Stage**				
**Test result**	**Normal**	**CIN 1**	**CIN 2,3**	**CC I**	**CC II-IV**
P1-2	0.31	0.1	0.04	0.01	0
P3	0.621	0.45	0.48	0.04	0
P4-5	0.069	0.45	0.48	0.95	1

CIN: cervical intraepithelial neoplasia, CC: cancer, P: Papanicolaou.

### Vaccination

Three different vaccination strategies can be modeled: no vaccination, vaccination at age 12 with lifetime protection assumed, and vaccination at age 12 plus booster vaccination 20 years later assuming a waning of the vaccine efficacy after 20 years.

The efficacy of vaccination was modeled as the product of the proportion of cervical cancers caused by the oncogenic HPV types that the vaccine provides protection against and the vaccine efficacy against a 6-month persistent infection with these types of HPV. For the former, 71.5% was used as the default value because that is the proportion of cervical cancers that can be attributed to HPV types 16 and 18 in Europe [12]. The vaccine efficacy against persistent HPV 16/18 infection was modeled as 94.3% [13]. We took into account cross-protection against other types of HPV in the sensitivity analysis of the model because the vaccine provides significant protection not only for HPV types 16 and 18 but also for HPV types 33, 31 and 45 (Table 4) [14]. Although the model did not directly distinguish the different high risk HPV types, this way we could model the efficacy considering different HPV types. Additionally, we performed sensitivity analysis with the intention-to-treat estimate of the efficacy parameter, which was 90.2% in all vaccinated subjects who had received at least one dose, for whom data concerning efficacy endpoint measures were available and who had a normal or low-grade cytology (defined as negative, or ASCUS or LSIL) at month 0 [13]. The incidence among vaccinated persons was assumed to be decreased by the vaccine efficacy.

**Table 4:** Cervical cancers attributable to different types of HPV and protection against 6-month persistent infections by the AS04-adjuvanted bivalent vaccine

**HPV type**	**Cervical cancers attributable to the type (%)**	**Vaccine efficacy (%)**	**Vaccine efficacy against oncogenic infection (%)**	
			**Without cross-protection**	**With cross-protection**
16	65.4	94.3	67.42	75.33
18	6.1			
33	5.6	45.1		
31	4.1	77.5		
45	2.9	76.1		

Current evidence suggests long-term vaccine efficacy [14,15]. Mathematical modeling predicts that the AS04-adjuvanted HPV 16/18 vaccine provides long-lasting high antibody levels against HPV types 16 and 18 [16,17]. Based on the current evidence, we did not assume waning immunity in the base; nevertheless, in the sensitivity analysis, we assessed the cost-effectiveness assuming that the vaccine efficacy decreases to 50% from 20 to 30 years after vaccination and a booster is given 20 years after the first immunization in 80% of women vaccinated at the age of 12.

Vaccination coverage can be set for both the initial vaccination and for the booster dose as a percentage of the cohort; the base case values were 80% for the former and 0% for the latter. Vaccination coverage had relevance if herd immunity was taken into account. Herd immunity was modeled by applying an additional reduction in the incidence rate from state “No CIN, HPV negative” to the state “No CIN, HPV positive”. The incidence rate applied was inversely related to the vaccination coverage rate and the vaccine efficacy. The incidence rate in the population was estimated as the weighted average of the expected incidence among non-vaccinated and vaccinated persons, where the weights were the proportions of these subpopulations. Herd immunity was taken into account in the base case scenario. If the vaccination coverage did not reach 30%, the model did not take into account the herd immunity effect.

For the base case, no decrease in the quality of life due to the vaccination was assumed.

### Cost data

The cost of the screening process and cancer treatment was taken from a previously published model (Table 5) [7]. The nominal prices paid by the National Sick Fund for the units of in- and outpatient services have increased by 2.74% since the cost estimation was performed for the previous model. Therefore, we updated the cost data accordingly. The initial model included costs in Hungarian forints. These costs were converted to US$ based on the purchasing power parity exchange rate of 129 HUF/$ (2010) to eliminate differences in price levels between countries [18]. Resource utilization data of the screening process was estimated per protocol: resource units were multiplied by the tariffs of the National Health Insurance Fund for the services. The costs of the Pap-smear (National Health Insurance Fund (NHIF) procedure code: 14720), the cytological examination (NHIF procedure code: 42700) and of the gynecological screening examination (NHIF procedure code 16631 plus 42600) were $0.73, $12.7 and $12,12, respectively. The price of local anti-inflammatory treatment was $6.24. The cost of the conisation was $1,495.2. The cost of conisation was calculated by the cost of the diagnosis-related group “operation of uterus and adnexum of uterus due to in situ carcinoma and non-malignant disease” coded as 643B.

**Table 5:** Direct medical cost ($) per month of cervical cancer by stages and time after the diagnosis

	**Time after the diagnosis**			
**Stage**	**Months 0-3**	**Months 4-12**	**Months 13-24**	**From month 25**
CC I-IIa	1 157 (289.20)	216 (54.05)	158 (39.39)	99 (24.72)
CC IIb-III	1 157 (289.20)	353 (88.51)	339 (84.54)	283 (70.56)
CC IV	1 157 (289.20)	352 (87.90)	447 (111.82)	328 (81.98)

The numbers in brackets are the standard errors applied for the distributions in the probabilistic sensitivity analysis.

CC: cancer.

We used the European Economic Area’s lowest official public price of the vaccine, $131.08, in the analysis because if the vaccination is included in the national vaccination scheme in Hungary as a 100% reimbursed vaccine, then its price cannot be higher than that because of reference pricing rules. Three doses of the vaccine are required for the basic immunization.

We did not consider indirect costs in the analysis.

### Sensitivity analysis

We tested the robustness of the results to 10% increases and decreases in the input parameters. In the probabilistic sensitivity analysis, we defined distributions for the key input parameters and ran 5000 Monte-Carlo simulations with sampling from these distributions. Gamma distributions were applied to the incidence rates of transitions, the participation rates, the cancer stage-specific mortality, the stage-specific health care costs and the organizational costs of screening. Ten percent of the point estimates was used as standard error of the parameters. Beta distributions were applied to the screening participation rates and the quality-of-life weights of the cancer states. Twenty-five percent of the point estimates was used as standard error of the parameters. Dirichlet distribution was used for the sensitivity and specificity of the screening test. We did not use standard errors for the parameters with Dirichlet distribution. We plotted the results on an incremental cost-effectiveness ratio scatter plot and on an acceptability curve to determine the proportion of the simulated results that were below the cost-effectiveness threshold.

## Results

Table 6 shows the costs and the QALYs corresponding to the vaccination and no-vaccination strategies. The incremental cost-effectiveness ratio of vaccination was 27 588 $/QALY. Table 7 shows that the cost-effectiveness of vaccination was very sensitive to some of the key input parameters, including the price of the vaccine, the discount rate and whether herd immunity was taken into account. Without herd immunity, the ICER changed from 27 588 to 42 520 $/QALY. With the use of the intention-to-treat vaccine efficacy parameter, the ICER slightly increased to 28 662 $/QALY. When cross-protection was taken into account, the ICER decreased to 25 190 $/QALY. Assuming that the vaccine efficacy waned after 20 years, requiring the administration of a booster dose, largely decreases the cost-effectiveness.

**Table 6:** Estimated cost-effectiveness of the different strategies

**Strategy**	**QALY**	**Cost ($)**	**ICER ($/QALY)**
no vaccination	22.427	3 348.8	reference
vaccination	22.437	3 629.2	27 588

QALY: quality-adjusted life years, ICER: incremental cost-effectiveness ratio.

**Table 7:** The effect of major input parameters on the estimated cost-effectiveness of adding vaccination to screening

	**ΔQALY**	**Δcost ($)**	**ICER ($/QALY)**
base case	0.01016	280.396	27 588
no herd immunity	0.00857	364.382	42 520
with intention-to-treat efficacy parameter	0.00982	281.534	28 662
with cross-protection	0.01102	277.560	25 190
50% waning of efficacy from 20 to 30 years after vaccination and booster dose in 80% of persons immunized at age 12 years and alive after 20 years	0.00728	332.731	45 709
discount rate 5%	0.00532	295.598	55 617
price of the vaccine 231$*	0.01016	520.210	51 184

*current recommended market price of the Cervarix vaccine in Hungary.

QALY: quality-adjusted life years, ICER: incremental cost-effectiveness ratio.

The results of the detailed deterministic and the probabilistic sensitivity analyses are presented in the online Additional file 2.

## Discussion

Our study aimed to provide health economic data to inform the development of the Hungarian cervical cancer prevention program. First, the national program needs to address the problem of the inefficiency of the current screening program [5,6]. We have previously developed a health economic model to support this decision making process [7]. Furthermore, with the introduction of vaccination against cervical cancer, a new policy question was raised: whether to include the vaccination of adolescent girls in the cervical cancer prevention program. We aimed to analyze this question from a health economic perspective.

Our modeling results predicted that adding vaccination of adolescent girls with the AS04-adjuvanted human papillomavirus 16/18 vaccine to the national cervical cancer screening program would be cost-effective in Hungary. Although our results were quite robust to the uncertainty in the input parameters, larger changes in the most influential parameters could considerably change the result.

An important question from the policy perspective is whether a catch-up vaccination for older girls/women would be beneficial and cost-effective. Studying this question was outside the scope of our analysis because catch-up vaccination against cervical cancer is not under consideration in the Hungarian health policy agenda.

Our analysis can be considered a conservative one because we did not consider potential health benefits of vaccination other than preventing cervical cancer, including protection against other diseases. This lack of consideration of other potential benefits is true for the screening test as well. Some gynecological abnormalities can be detected when the cervical smear is taken. Furthermore, we did not include indirect costs in our analysis. Another limitation of our study is that the reported cost-effectiveness can be expected only after a few years time because it is necessary for the incidence of HPV among the partners of an actually vaccinated population to decrease for herd immunity to develop. Furthermore, we did not use a dynamic transmission model, and did not model the incidence of the different HPY types. Therefore, our method of modeling the effect of the herd immunity can be considered a simple tool to project the main features of the potential impact of HPV vaccines at the population level. Additionally, our model was somewhat undercalibrated in the young age groups, and it was well calibrated only above age 44 years, i.e. the number of the estimated new cases with the current screening practice and no vaccination was consistent with the actual incident cases registered in the National Cancer Registry above age 44 years [7].

Many modeling studies have been published that investigated the cost-utility of adding HPV vaccination to existing screening programs in different countries [19-36]. The direct comparison of the results is not possible, because of the differences in the methods and because of the differences in the input parameters. The studies themselves and their reviews highlighted the limitations of the transferability of health economic evaluations [37-42]. Most of the variations can be explained by the differences in the model structures (e.g., modeling herd immunity or not) and in the influential input parameters (e.g., the incidence of cervical cancer related to the effectiveness of the screening, the cost of the vaccine, waning of the vaccine efficacy, the use of booster vaccination, and the value of the discount rate). The huge effect of the discount rate on these published results and on our results highlights the challenges involved in the health economic analysis of primary preventive programs. In these programs, the return of an early investment can be expected in the long run in the form of improved health; thus, the discount hardly affects the cost, but it devalues the benefit.

A cost-effectiveness analysis of the quadrivalent (6/11/16/18) HPV vaccine in Hungary has already been published [43]. The authors reported that the incremental cost-effectiveness ratio of adding vaccination at age 12 years to the current screening program was 9 577 €/QALY, which is equivalent to $18 901 when using the €/HUF exchange rate given the authors used and the purchasing power parity exchange rate of 129 HUF/$. In addition to the differences between the vaccines studied, the major methodological differences between studies make it difficult to compare our results with those of that analysis. First, our model tracks a cohort, whereas the other model follows a population of fixed size at any time point. Hence, the compositions of the numerators and denominators used in the ICERs differ between the models. Second, the results of the other study accounted for the additional benefits conferred by protecting against HPV 6/11 infection. Finally, the states that were modeled and the parameters applied differed as well.

## Conclusions

Our model predicted that adding vaccination of 12-year-old adolescent girls with the AS04-adjuvanted human papillomavirus 16/18 vaccination to the national cervical screening program would be a cost-effective strategy to prevent cervical cancer in Hungary.

## Abbreviations

HPV: Human papillomavirus; QALY: Quality-adjusted life years; QoL: Quality-of-life.

## Competing interests

This work was financially supported by GlaxoSmithKline Hungary, Ltd. GlaxoSmithKline manufactures Cervarix^TM^.

## Authors’ contributions

All of the authors participated in the design of the study. ZV developed the structure of the model. LN and ZV collected the data on the input parameters. LN performed the programming. ZK supervised the work and tested and further developed the model. ZV drafted the first version of the manuscript. All authors participated in the revision of the manuscript and approved the final version.

## Pre-publication history

The pre-publication history for this paper can be accessed here:

http://www.biomedcentral.com/1471-2458/12/924/prepub

## Supplementary Material

Additional file 1 **Age specific incidence rates of the transitions from one state to another. **MS-Excel format. HPV: human papilloma virus, CC: cancer, CIN: cervical intraepithelial neoplasia. Click here for file

Additional file 2 **Results of the deterministic and probabilistic sensitivity analyses. Figure 1:** Sensitivity of the cost-effectiveness to 10% increases and decreases in the most influential input parameters. **Figure 2:** Cost-effectiveness acceptability curve. Click here for file
