# Supplementary material for: Cost-effectiveness of adding vaccination with the AS04-adjuvanted human papillomavirus 16/18 vaccine to cervical cancer screening in Hungary
Source: BMC Public Health. 2012 Oct 30;12:924. doi: 10.1186/1471-2458-12-924 (PMC3528422; doi:10.1186/1471-2458-12-924)
Supplement: Additional file 2 — Results of the deterministic and probabilistic sensitivity analyses. Figure 1: Sensitivity of the cost-effectiveness to 10% increases and decreases in the most influential input parameters. Figure 2: Cost-effectiveness acceptability curve. [file 1471-2458-12-924-S2.doc]

**Additional file 2 - Results of the deterministic and probabilistic sensitivity analyses**

Additional Figure 1 shows how much a 10% increase or decrease of the 10 most influential parameters changed the ICER. Δcost varied between 151.7 and 407.9 $, and ΔQALY varied between 0.00936 and 0.01089. The largest increase in the ICER was 12 534 $ / QALY, which was observed in response to a 10% increase in the proportion of vaccinated women starting screening at the age of 18, and the largest decrease was 12 338 $ / QALY, which was observed in response to a 10% decrease in the proportion of vaccinated women starting screening at age 18. Additional Figure 2 shows that our result is quite robust relative to the stochastic uncertainty of the input parameters. The variation of results of the 5000 simulations is reasonably small. Δcost had a range between 228.8 and 340.4 $, and ΔQALY had a range of 0.007294 to 0.013385. The ICER had a range of 20 409 to 39 362 $/ QALY, and all of the simulated ICERs were below the informal Hungarian cost-effectiveness threshold of 55 800 $ / QALY (Additional Figure 2).

# Figure legend

## Additional Figure 1 - Sensitivity of the cost-effectiveness to 10% increases and decreases in the most influential input parameters

CC: cancer, QoL: quality of life, ΔICER: change in the incremental cost-effectiveness ratio, QALY: quality-adjusted life years

## Additional Figure 2 - Cost-effectiveness acceptability curve

QALY: quality-adjusted life years
